# Multi-cancer blood testing combined with PET-CT: road for hope to screen for cancer and guide intervention

**DOI:** 10.1038/s41392-020-0210-2

**Published:** 2020-06-12

**Authors:** Tao Jiang, Shengxiang Ren, Caicun Zhou

**Affiliations:** Department of Medical Oncology, Shanghai Pulmonary Hospital & Thoracic Cancer Institute, Tongji University School of Medicine, Shanghai, China

**Keywords:** Genetics, Tumour biomarkers

**Fig. 1:**
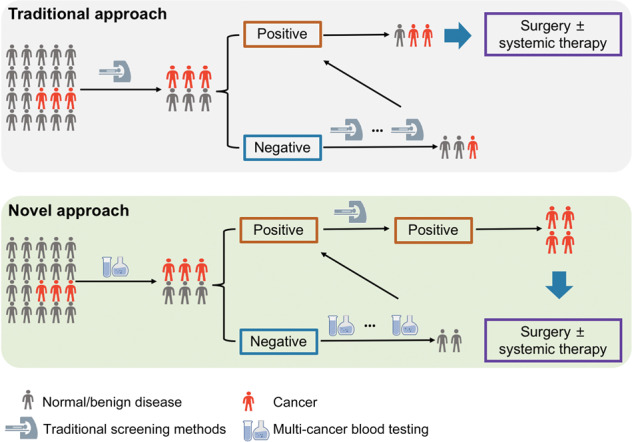
Traditional vs. novel cancer screening approach. Novel cancer screening approach would integrate the multi-cancer blood testing into traditional standard-of-care strategies

**In a recent study published in*Science*, Lennon et al.**^1^**investigated the feasibility and safety of multi-cancer peripheral blood testing in combination with positron emission tomography-computed tomography (PET-CT) imaging to detect cancer in 10,006 women not previously known to have cancer. Their findings reveal that multi-cancer blood testing coupled with diagnostic PET-CT can be safely incorporated into routine clinical care to screen for cancer without discouraging patients from engaging in other forms of standard-of-care (SOC) screening; moreover, it is possible to intervene on the basis of blood testing results, in some cases leading to surgery with intent to cure (Fig.** **1****).**

Most cancers could be cured by surgical resection without any systemic treatment at an earlier stage.^2^ In spite of the recent development and progress in surgery, radiation, and conventional as well as novel therapeutic agents, including immunotherapy, the majority of advanced or metastatic cancers would be incurable. Therefore, effective screening and earlier detection is the key to reducing cancer-related mortality. In fact, several screening modalities, including low-dose computed tomography, colonoscopy, mammography, and Pap smears, have been incorporated into routine clinical care and demonstrated to decrease mortality from lung, colon, breast, and cervical cancers, respectively.^3^ However, low sensitivity and/or specificity of current SOC screening and finite screening approaches for other cancers are the major dilemmas in this area. With the rapid development of gene sequencing and liquid biopsy techniques, peripheral blood testing is considered as one of the most exciting advances in cancer diagnostics. Several publications have shown promising results to identify cancers through blood testing at an early stage.^4^ Nevertheless, the sensitivity of blood testing would be very low when applied to patients not already known to have cancer. Considering the effectiveness of current SOC screening approaches, an ideal and novel multi-cancer blood test should increase cancer detection rates in a complementary way to SOC screening approaches.

To evaluate the feasibility and safety of a novel multi-cancer blood testing (a multi-analyte peripheral blood test, including DNA and protein biomarkers) in combination with PET-CT, Lennon et al.^1^ performed an exploratory prospective, interventional study named DETECT-A (Detecting cancers Earlier Through Elective mutation-based blood Collection and Testing) to enroll 10,006 women (age range 65–75 years) not already known to have cancer, but with high adherence to SOC screening. In this study, 96 cancer diagnoses were made, including 26 detected by multi-cancer blood testing, 24 detected by SOC screening, and 46 by neither approach. Of 26 cancers detected by blood testing, 17 were localized or regional and 9 were surgically resected. Only 1.0% of participants underwent PET-CT imaging based on false-positive blood tests, and 0.22% received an unnecessary invasive diagnostic procedure. Given the promising feasibility and safety of blood testing in combination with diagnostic PET-CT imaging in this study, it is valuable to design and conduct future randomized, interventional trials to investigate the ability of minimally invasive multi-cancer blood tests to improve the effectiveness of cancer screening and early detection.

The DETECT-A is the first prospective and interventional study of this nature so that safety is the priority. To guarantee the safety of each participant, the authors conducted a series of safety features. First, participants were counseled and educated about interpretations of positive and negative tests and the need for continued SOC screening at enrollment. The abnormal original baseline blood test rigorously excluded clonal hematopoiesis of indeterminate potential (CHIP) and would be tested again for a distinct confirmation test. If the blood test was reproducibly abnormal in the confirmation test and CHIP was ruled out, it was considered positive. Second, the Multidisciplinary Review Committee (MRC) reviewed the medical history of participants to exclude a potential non-cancer-related cause for any abnormal results and tests would be relayed to participants in a careful and prescribed manner. Third, when the researcher excluded above-mentioned cause, the participants were suggested to receive diagnostic PET-CT imaging to confirm the blood testing results and localize the potential cancer. Fourth, patients with cancer were defined as those with biopsy-proven cancer or other undisputed clinical evidence of disease. The MRC recommended the follow-up after concerning PET-CT scans and continued SOC screening was recommended for all participants.

Between September 2017 and May 2019, 10,006 participants were enrolled and 9911 were eligible. Of them, 490 (4.9%) scored positive in the baseline blood test and 134 were confirmed in the confirmation test. Of the 134 participants, 127 were evaluated by diagnostic PET-CT imaging. Sixty-four of these 127 participants had imaging concerning for cancer. Twenty-six of 64 patients were subsequently shown to have cancer through biopsy or other undisputed clinical evidence. The sensitivity, specificity, and positive predictive value of testing combined with imaging were 27.1%, 99.6%, and 40.6%, respectively. Of 108 participants with positive blood testing but no cancer, 38 had any procedure subsequent to their PET-CT findings. Of 38 participants, only three cases received surgery. No study protocol-related serious adverse events were observed. Notably, in 6874 participants who completed a survey on their impression of this study, only 0.3% of them considered participating in the study as a wrong decision and 1.0% of respondents refused to join a similar, subsequent study if it were offered to them again.

Collectively, the DETECT-A affirmed that blood testing is possible for early detection of cancers, and to intervene based on blood testing, leading to surgery with intent to cure. Such blood testing can be incorporated into routine clinical care and performed in a safe manner without incurring a large number of futile, invasive follow-up tests. Considering the very short follow-up of this study, longitudinal follow-up for up to 5 years is needed. Additionally, it should be stressed that the DETECT-A study was not designed for regulatory approval of a specific blood test. Future trials are needed to accurately investigate benefit vs. risk, and the clinical validity and utility of such multi-cancer blood testing. Notably, the baseline blood test was only an early version of a multi-analyte test, called CancerSEEK. Recently, the updated version of CancerSEEK incorporating genetic and protein biomarkers showed sensitivities of 69–98% for the detection of five common cancer types (ovary, liver, stomach, pancreas, and esophagus) and specificity of >99%, suggesting that such multi-analyte blood test could help identify those patients most likely to harbor a malignancy.^5^ The future randomized, interventional trials need to further evaluate the ability of minimally invasive blood tests incorporating other cancer biomarkers, including messenger RNA transcripts, microRNAs, metabolites, or methylated DNA sequences to improve the effectiveness of cancer screening.
